# Use of the Hourglass peripheral embolisation device: early experiences

**DOI:** 10.1186/s41747-017-0035-0

**Published:** 2018-02-21

**Authors:** Peter Hughes, Ian Brennan, J. Mark Ryan

**Affiliations:** Department of Radiology, St. James’s Hospital, Dublin 8, Ireland

**Keywords:** Vascular plug, Embolisation, Hourglass, Arterial interventions, Venous interventions

## Abstract

We evaluated a novel intravascular plug, the Hourglass peripheral embolisation device (PED). We describe, for the first time, the use of this device and discuss its potential applications. The device was deployed in nine patients over a six-month period at a single institution by two different operators. Five patients underwent renal artery embolisation, three underwent gonadal vein embolisation for a varicocele, and a single patient underwent embolisation of the gastroduodenal artery. We recorded the indications, success rate, and the procedure-related complication rate in all patients. We also evaluated the satisfaction of the operators with the device using a post-procedure evaluation form. Technical success was achieved in 9/9 (100%) cases. Unanimous feedback was obtained from the operators (100% agreement). The usability of the delivery system, device deployment, and device visibility under fluoroscopy were rated as easy in 9/9 (100%) cases. The ease of repositioning was rated as good in both cases where this was attempted. The device trackability was rated as good in 9/9 (100%) cases. There were no procedure-related complications. The Hourglass PED is potentially useful for the embolisation of small-to-medium sized vessels.

## Key points

- The Hourglass device is a potentially useful option in the evolving field of endovascular plugs
- The device was deployed in nine patients with a 100% technical success rate and no complications
- Operator satisfaction with the device was high

## Background

The role of endovascular embolisation in both routine and emergent clinical practice continues to expand apace, along with an increase in the frequency and complexity of procedures being performed. Similarly, the array of embolic agents available to the interventional radiologist has expanded in recent years with the advent of novel vascular plug devices. The Amplatzer plug devices (St Jude Medical Inc., Saint Paul, MN, USA) are already well established [1–3]. Recent publications have shown promising results with the Microvascular Plug (Reverse Medical Corporation, Irvine, CA, USA) [4–6]. Endovascular coils are the current embolic agent of choice for occlusion of small-to-medium sized vessels; however, the variability in size and number of coils required to achieve target vessel occlusion is a major drawback of these agents. In contrast, vascular plugs allow target vessel occlusion with deployment of a single device thus shortening the procedure time, screening time and cost.

The Hourglass device is a novel vascular plug developed by EMBA Medical (Dublin, Ireland). The device was CE-marked for use in Europe in July 2015 and was approved for use by the Food and Drug Administration in August 2017. The device is an over-the-wire intravascular plug with a polytetrafluoroethylene (PTFE)-covered Nitinol frame which aims to provide immediate occlusion in small-to-medium sized vessels. To date, no published data exist on the use of this device. This paper describes a single-centre experience of embolisation for a variety of clinical indications using the Hourglass device in nine patients.

## Methods

### Patients, operators, and evaluation forms

A total of nine patients underwent embolisation using the Hourglass device. They were four females and five males (average age 52 years; age range 25–84 years). Five patients underwent renal artery embolisation, three had left gonadal vein embolisation for varicoceles and a single patient underwent embolisation of the gasttroduodenal artery (GDA) for a bleeding duodenal ulcer after failed endoscopic treatment.

The procedures were performed by two different primary operators with 22 years and 9 years of interventional radiology experience, respectively. A post-procedure evaluation form was filled out after each procedure by the primary operator. The primary operator was asked to grade satisfaction with the device in relation to the following attributes: usability of the delivery system; device deployment; device visibility under fluoroscopy; and ease of repositioning, if attempted. Each was graded on a three-point scale (easy, somewhat difficult or unsatisfactory). The stability and trackability were also scored on a three-point scale (good, fair or poor).

### Device

**Fig. 1:**
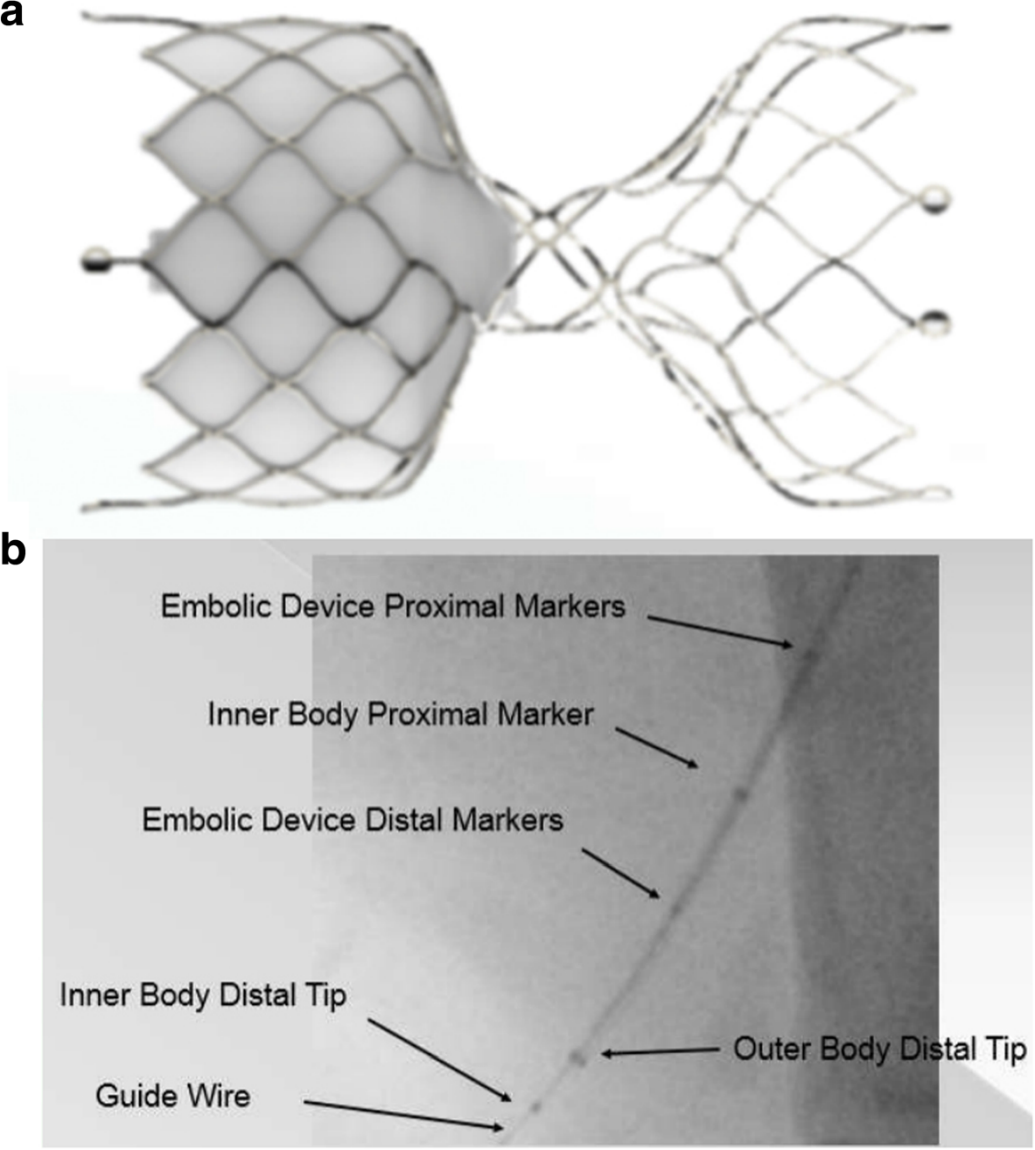
The EMBA Hourglass device: **a** A Nitinol frame, the proximal half of which is covered with PTFE. Unconstrained, the device measures 20 mm in length and 10 mm in diameter. **b** Single radiographic image showing the device and delivery system in situ with the location of the radio-opaque markers indicated

The Hourglass device comprises an hourglass-shaped Nitinol frame, the proximal half of which is covered with PTFE (Fig. 1). Unconstrained, the device measures 20 mm in length and 10 mm in diameter, although the final device length depends on vessel size. It is designed as a ‘one size fits all’ device for use in vessels with diameters in the range of 3–8 mm. The distal end of the device acts as an anchor, while the covered proximal end provides immediate occlusion of the vessel when deployed. The hourglass configuration aims to harness the effect of blood pressure to augment the compressive forces on the frame providing additional anchoring and occlusion.

The device is delivered over a 0.018” guidewire in a similar manner to an endovascular stent. The delivery system consists of an inner body, an outer body and a rotating haemostatic valve (RHV) with a side port. There are radiopaque markers on either end of the device, on the distal tip of the outer body and on the proximal tip and mid portion of the inner body.

### Deployment technique

**Fig. 2:**
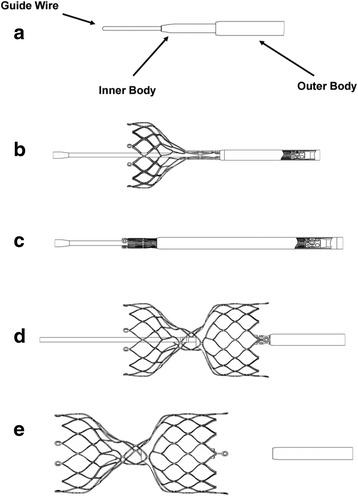
Illustrating step-by-step deployment of the device (**a**–**e**), described in detail in the main body of the text

The device was deployed in an identical manner in all cases (Fig. 2). A 0.018” wire was positioned within the target vessel across the desired location for deployment. The delivery system was then introduced and the distal tip placed just beyond the desired landing site. The inner body and embolic device were then advanced within the outer body until the distal end of the embolic device was aligned with the distal end of the outer body. The RHV on the inner body was then loosened and the device partially deployed by holding the inner body stationary and withdrawing the outer body in a “pinch-pull” manner (Fig. 2b). At this point, the device may be re-sheathed by re-advancing the outer body provided the outer body tip marker does not pass the proximal marker of the inner body (Fig. 2c).

Once the device was anchored distally, full deployment was achieved by pulling the outer body back to unsheath the proximal end of the device (Fig. 2d). Pushing slightly forward on the inner sheath disengaged the device allowing the inner body and outer body to be simultaneously withdrawn, while keeping the guide wire stationary (Fig. 2). Finally, the wire was withdrawn through the device. Contrast injection via the outer body side port immediately confirmed the position of the device and assessed the adequacy of vascular occlusion.

### Case illustrations

#### Renal artery embolisation

**Fig. 3:**
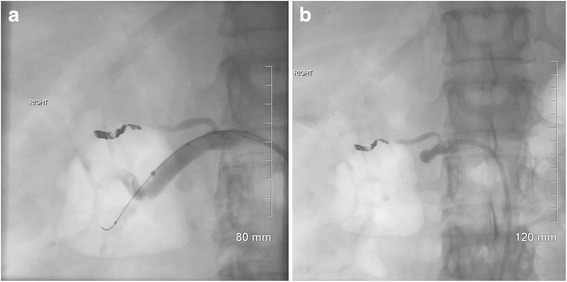
Digitally subtracted angiographic images demonstrating the right renal artery (**a**) before and (**b**) after deployment of the device. Note the immediate cessation of flow in the target vessel. Also visible are additional coils in a proximal branch to the right upper pole

The procedure was carried out in five patients (A–E) via a right common femoral artery approach using a 6-Fr Destination sheath (Terumo Europe, Leuven, Belgium) placed in the proximal renal artery and a V-18 guidewire (Boston Scientific, Marlborough, MA, USA). Patients A and B had renal cell carcinomas which required de-vascularisation before surgical resection. Patient C underwent embolisation of a renal mass without subsequent surgery. Patient D developed a perinephric haemorrhage around a non-functioning left kidney on a background of prior transplant and anticoagulant medication. Patient E had a non-functioning left kidney with severe hydronephrosis. In all five patients, distal target organ embolisation was first performed using embospheres (Merit Medical Systems, South Jordan, UT, USA) before deployment of the plug. Patient A required additional coil embolisation of an upper pole branch arising proximally from the left renal artery (Fig. 3) and patient D required coil embolisation of the right adrenal artery.

#### Varicocele embolisation

**Fig. 4:**
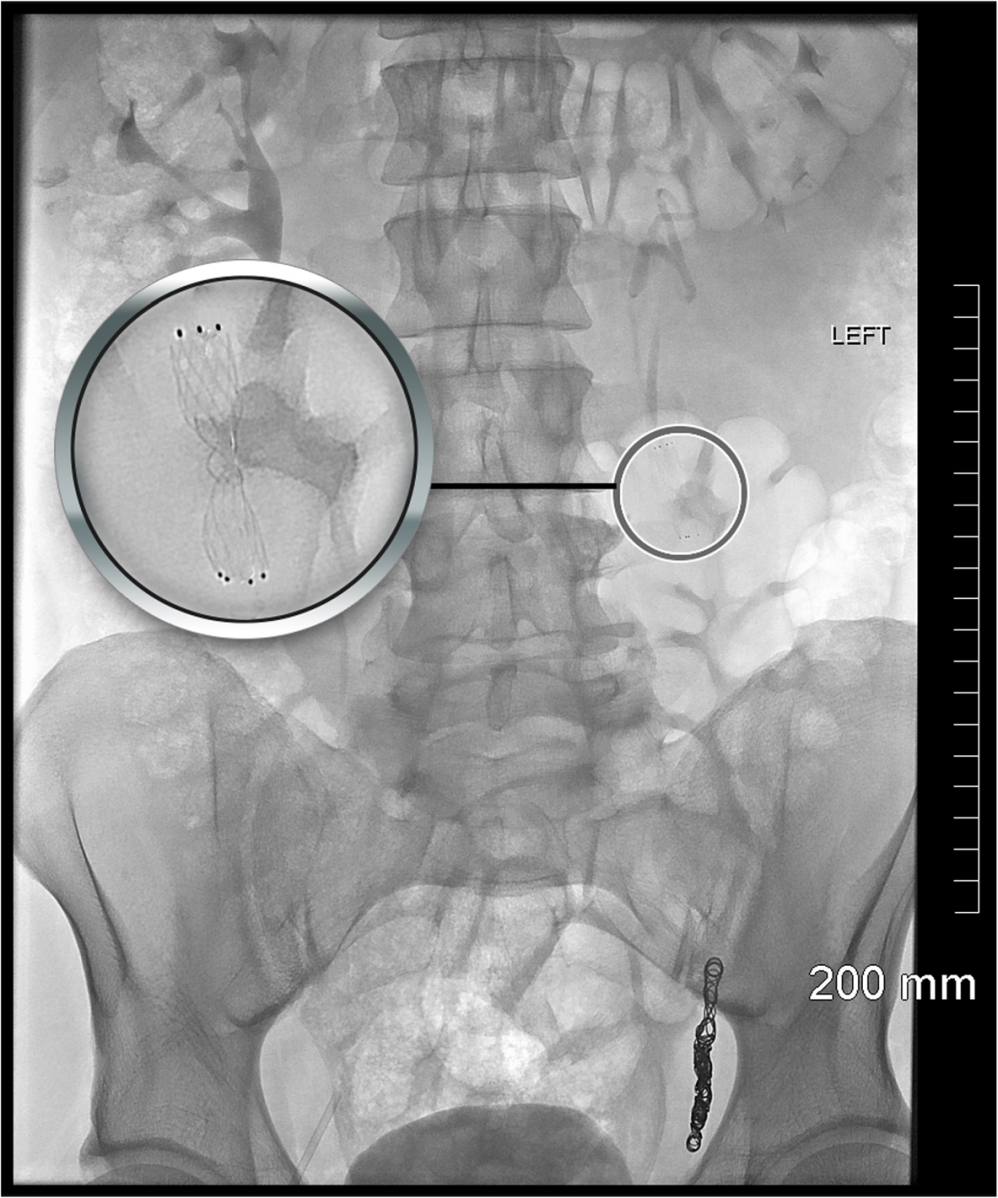
Single radiographic image with magnification over the device in situ within the left gonadal vein

All three patients (F–H) had symptomatic left sided varicoceles and underwent embolisation of the left gonadal vein. A right common femoral vein approach was used and a C2 catheter (Cook Medical, Bloomington, IN, USA) used to catheterise the left renal vein. The gonadal vein was selected using a hydrophilic guide wire and the C2 catheter advanced into the proximal portion. This was then exchanged for a 6-Fr sheath, which was advanced into the proximal left gonadal vein. Retrograde venography confirmed a dilated and incompetent left gonadal vein in all three cases. The vein was embolised with coils distally and the Hourglass device was deployed proximally (Fig. 4).

#### Gastroduodenal artery embolisation

One patient (I) presented with an upper gastrointestinal haemorrhage arising from a duodenal ulcer, confirmed with direct visualisation. Endoscopic attempts to control the haemorrhage were unsuccessful. The patient subsequently underwent GDA embolisation, using coils for the ‘back door’ supply from the superior mesenteric artery and the Hourglass device in the proximal GDA.

## Results

In 9/9 (100%) cases, the device was successfully deployed with immediate, angiographically proven occlusion of the target vessel. There was no observed foreshortening of the device and there were no instances of device migration. The average time taken to deploy the device was 5 min (range = 3–10 min).

Both operators rated the device as easy to use and deploy in 9/9 (100%) cases. Repositioning of the device was performed twice and was rated as easy in both cases. The device was easily visible under fluoroscopy in 9/9 (100%) cases. The trackability and stability of the delivery system was rated as good in 9/9 (100%) cases.

**Fig. 5:**
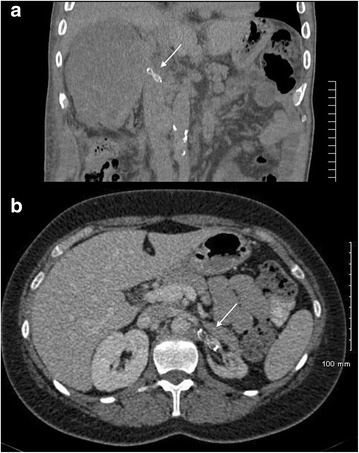
**a** Coronal image of an unenhanced CT showing the device (*white arrow*) in situ in the right renal artery. **b** Axial contrast-enhanced CT image from another patient showing the device (*white arrow*) in situ in the left renal artery

**Fig. 6:**
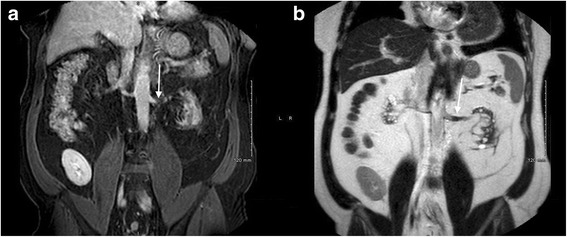
**a** Coronal MR angiography demonstrating an abrupt cessation of flow in the left renal artery (*white arrow*). **b** Coronal T1-weighted MRI from the same patient showing the device (*white arrow*) in situ within the left renal artery. Note the lack of susceptibility artefact

Patient outcomes were as follows: patient A died from complications related to the subsequent surgery; patient B underwent successful resection of the kidney one day after embolisation; patient C underwent follow-up non-contrast computed tomography (CT) of the abdomen, which showed the device in situ and necrosis of the tumour (Fig. 5a). Patient D had follow-up magnetic resonance imaging (MRI) one year later, which showed resolution of the perinephric haematoma (Fig. 6). Patient E had a follow-up CT which demonstrated the device in situ, marked cortical atrophy, and resolution of the hydronephrosis (Fig. 5b). All three patients who underwent varicocele embolisation reported symptomatic relief following the procedure. A follow-up CT angiogram one day post GDA embolisation confirmed satisfactory occlusion of the GDA with no evidence of extravasation.

## Discussion

This is the first published evaluation of the Hourglass device, a novel over-the-wire vascular plug delivered in the same manner as an endovascular stent – a familiar technique for interventional radiologists. The operators achieved a 100% technical success rate, scored the device as easy to use and reported a high level of satisfaction with both the device and the delivery system.

As described by Lubarsky et al. [7], the choice of embolic agent is based on the vessel size, the desired duration of embolisation and the need to maintain end organ viability. Vascular plugs are designed for permanent vessel occlusion and the use of a single device allows for rapid, accurate deployment compared with coils. A range of vascular plugs are available to suit the desired vessel characteristics; however, the operator must be aware of the differences in these devices in order to choose appropriately. The Hourglass device is a ‘one size fits all’ option, allowing deployment of a single device in vessels with diameters in the range of 3–8 mm. This has the potential to simplify procedures and reduce the range of vascular plugs required in stock.

We encountered no incidence of device migration or foreshortening in our study. While there were other embolic devices used for more distal embolisation in our patient cohort (i.e. endovascular coils and embospheres), the covered proximal end of the device was seen to result in immediate occlusion of the target vessel at the site of device deployment in all cases. The ability to inject contrast through the outer body side port allowed immediate confirmation of this target vessel occlusion. In theory, this could provide an advantage over other plugs whose interstices permit continued antegrade flow for a time following deployment; this results in a lower efficiency of occlusion and a variable and unpredictable time to occlusion [1, 3, 8]. Persistent patency has also been described as an issue with respect to other plugs, although this is less than with coil embolisation and usually in absence of angiographically demonstrable vessel occlusion at deployment [1–3]. In our study, immediate vascular occlusion was achieved and demonstrated in all cases.

The delivery system has an outer diameter of 1.67 mm allowing deployment through a 5-Fr catheter/sheath. The trackability of the delivery system was always rated as good, which is encouraging since this has been described as an issue with other vascular plugs [1]. The device was fully retracted and repositioned on two occasions without difficulty. We also noted a lack of device-related artefacts on subsequent CT and MRI, compared with the relevant artefact often encountered with endovascular coils.

One overall criticism of the device was reported by the operators. The length of the wire (300 cm) and the length of the delivery system (120 cm) provided with the device were felt to be excessive and inconvenient. However, a new 80-cm delivery system has since obtained a CE mark (March 2017), which may overcome some of these difficulties.

We acknowledge that this is a small case series at a single institution with a heterogeneous patient population and includes both arterial and venous interventions. Despite this, the authors believe that the outcomes of this feasibility study warrant larger-scale evaluation of the device.

In conclusion, the EMBA Hourglass device is a novel endovascular plug with several potential advantages over existing devices. The device was found to be easy to use, with a high level of operator satisfaction. It resulted into immediate, angiographically proven vascular occlusion at the time of the procedure. Its versatility allows the use of a single device in a range of vessel sizes and the delivery system allows for accurate deployment. No procedure-related complications were experienced in our cohort of patients. Larger studies will be required to fully evaluate the usefulness of this device and to confirm these early promising outcomes.

## Abbreviations

CT: Computed tomography
GDA: Gastroduodenal artery
MRI: Magnetic resonance imaging
PED: Peripheral embolisation device
PTFE: Polytetrafluoroethylene
RHV: Rotating haemostatic valve
